# PRISM 2.0: A Technology System to Support Resource Access and Social and Cognitive Engagement

**DOI:** 10.1093/geroni/igab046.1191

**Published:** 2021-12-17

**Authors:** Sara Czaja, Walter Boot, Neil Charness, Wendy Rogers, Joseph Sharit

**Affiliations:** 1 Weill Cornell Medicine/Center on Aging and Behavioral Research, New York, New York, United States; 2 Florida State University, Tallahassee, Florida, United States; 3 University of Illinois Urbana-Champaign, Champaign, Illinois, United States; 4 Department of Industrial Engineering, University of Miami, Coral Gables, Florida, United States

## Abstract

Social isolation and lack of engagement are common among older adults and present a risk for emotional, physical and cognitive decline. Technology offers the potential of remediating these risks and enhancing opportunities for connectivity. In this paper we present an overview of the PRISM 2.0 multi-site RCT, which evaluated a simple to use Personalized Reminder Information and Social Management System (PRISM) among a sample of two hundred and forty-eight adults age 65+ in diverse contexts (Rural Locations, Assisted Living Communities and Senior Housing). PRISM 2.0 is a tablet-based system, intended to provide support for access to resources and information, new learning, social and cognitive engagement, and memory. We describe the goals and content of PRISM, the user-centered design process, and measurement strategies. We also discuss the challenges of conducting the trial during the COVID-19 pandemic and the strategies used to adapt the trial protocol within the three contexts.

